# Pleistocene Sea‐Level Fluctuation Shapes Archipelago‐Wide Population Structure in the Critically Endangered Lord Howe Island Cockroach *Panesthia lata*

**DOI:** 10.1002/ece3.72760

**Published:** 2026-03-02

**Authors:** Maxim W. D. Adams, Kyle M. Ewart, Nicholas Carlile, Harley A. Rose, James A. Walker, Ian Hutton, Simon Y. W. Ho, Nathan Lo

**Affiliations:** ^1^ School of Life and Environmental Sciences University of Sydney Sydney New South Wales Australia; ^2^ Ecosystems and Threatened Species Department of Climate Change, Energy, the Environment and Water Parramatta New South Wales Australia; ^3^ Australian Government Department of Agriculture Water and Environment Canberra Australian Capital Territory Australia; ^4^ Lord Howe Island Museum Lord Howe Island New South Wales Australia

**Keywords:** conservation genetics, island biogeography, Lord Howe Island, phylogenetics

## Abstract

The Lord Howe Island Group (LHIG) is one of Australia's most renowned archipelagos. Although a number of studies have investigated the biogeography and genetic diversity of species on Lord Howe Island (LHI) itself, the evolutionary distinctiveness of populations across LHI's satellite islets remains poorly understood. In this study, we explored the genetic structure and health of the Endangered, endemic cockroach *Panesthia lata* across four islands of the LHIG, using a panel of nuclear single‐nucleotide polymorphisms and complete mitochondrial genomes. Our analyses reveal that the lineage on the permanently isolated islet Ball's Pyramid is highly divergent from the remaining populations, while those on the episodically connected LHI, Roach Island, and Blackburn Island may have diverged as recently as the end of the last interglacial period. These results offer the first evidence that Pleistocene sea level lowstands allowed for historical faunal connectivity across the LHIG. Further, while *P. lata* was believed to have been locally extirpated by rodents on LHI, we discovered a surviving, relict population, albeit with high pairwise kinship indicative of a strong population bottleneck. We also detect relatively high levels of kinship in the other populations, suggesting potential inbreeding that could necessitate ongoing management. Finally, the combination of shallow genetic structure and low diversity suggests that genetic rescue from another island may be a viable strategy to conserve the LHI population of *P. lata* , as well as other species that have been similarly impacted by rodents.

## Graphical abstract

We demonstrate that populations of the critically endangered Lord howe Island cockroach Panesthia lata were connected by land bridges during the last glacial period. Its 4 isolated populations are now highly inbred, suggesting that conservation translocation may be a viable strategy for conservation.

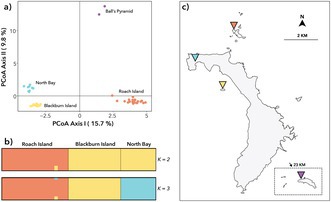

## 1 Introduction

The Lord Howe Island Group (LHIG) is a UNESCO World Heritage‐listed archipelago, recognised for its rich biodiversity. Occurring *ca*. 600 km east of mainland Australia, the LHIG comprises the eroded remnants of a large shield volcano that formed *ca*. 7 million years ago (Gilmore et al. 2020). The species richness of the LHIG is unusually high for an archipelago of its size and age, with surveys documenting 241 species of vascular plants, nearly 200 birds and over 1600 terrestrial invertebrates (Cassis et al. 2003; Green 1993; Lillemets and Wilson 2002; McAllan et al. 2004). The islands are considered major conservation priorities by Australian state and federal authorities (DECC 2007), and are presently managed under a Permanent Park Preserve.

Over 95% of the archipelago's land area is represented by Lord Howe Island (LHI) itself, a mountainous island covered predominantly by rainforest (Sheringham et al. 2016). The remaining terrestrial habitat is divided between 27 smaller islets, which sustain comparatively xeric ecosystems dominated by grasses and sedges (Carlile and Priddel 2013a, 2013b, 2013c, 2013d, 2013e; Carlile et al. 2013). Compared with LHI proper, the islets have received little scientific attention; in fact, most have never been formally surveyed. Consequently, many outstanding questions remain regarding the size, genetic distinctiveness and evolutionary history of the different islands’ faunal populations.

Across the globe, the evolution of many archipelagic biotas has been impacted by Pleistocene sea‐level fluctuation. During ancient periods of lower sea level (‘lowstands’), many presently isolated islands amalgamated into larger ‘palaeo‐islands’, permitting overland migration and gene flow among resident organisms. As a result, contemporary populations inhabiting fragments of a single palaeo‐island are often less evolutionarily distinct than those divided across permanently separated land masses, with genetic structure as shallow as the most recent lowstand (terminating ca. 10 ka; e.g., Barker et al. 2012; Fattorini 2010; Heaney et al. 2005; Papadopoulou and Knowles 2017). However, this pattern is far from universal, since the extent of overland migration depends on the unique ecological and geological conditions of each island system, as well as the vagility of the resident organisms. Many studies have found little to no effect of palaeo‐island arrangement on genetic structure, in archipelagos comparable to or larger than the LHIG (albeit most commonly in volant taxa such as birds; e.g., Choueri et al. 2017; Cros et al. 2020; Cros and Rheindt 2017; Garg et al. 2018; Lavery et al. 2023; Rijsdijk et al. 2014). Nonetheless, there are several examples of low genetic structure across paeleo‐islands in less vagile taxa such as insects and reptiles (Myers et al. 2025; Papadopoulou and Knowles 2015; Siler et al. 2010; Strehl and Gadau 2004; but see Fattorini 2010; Jordan et al. 2005; Reynolds et al. 2017).

**FIGURE 1:**
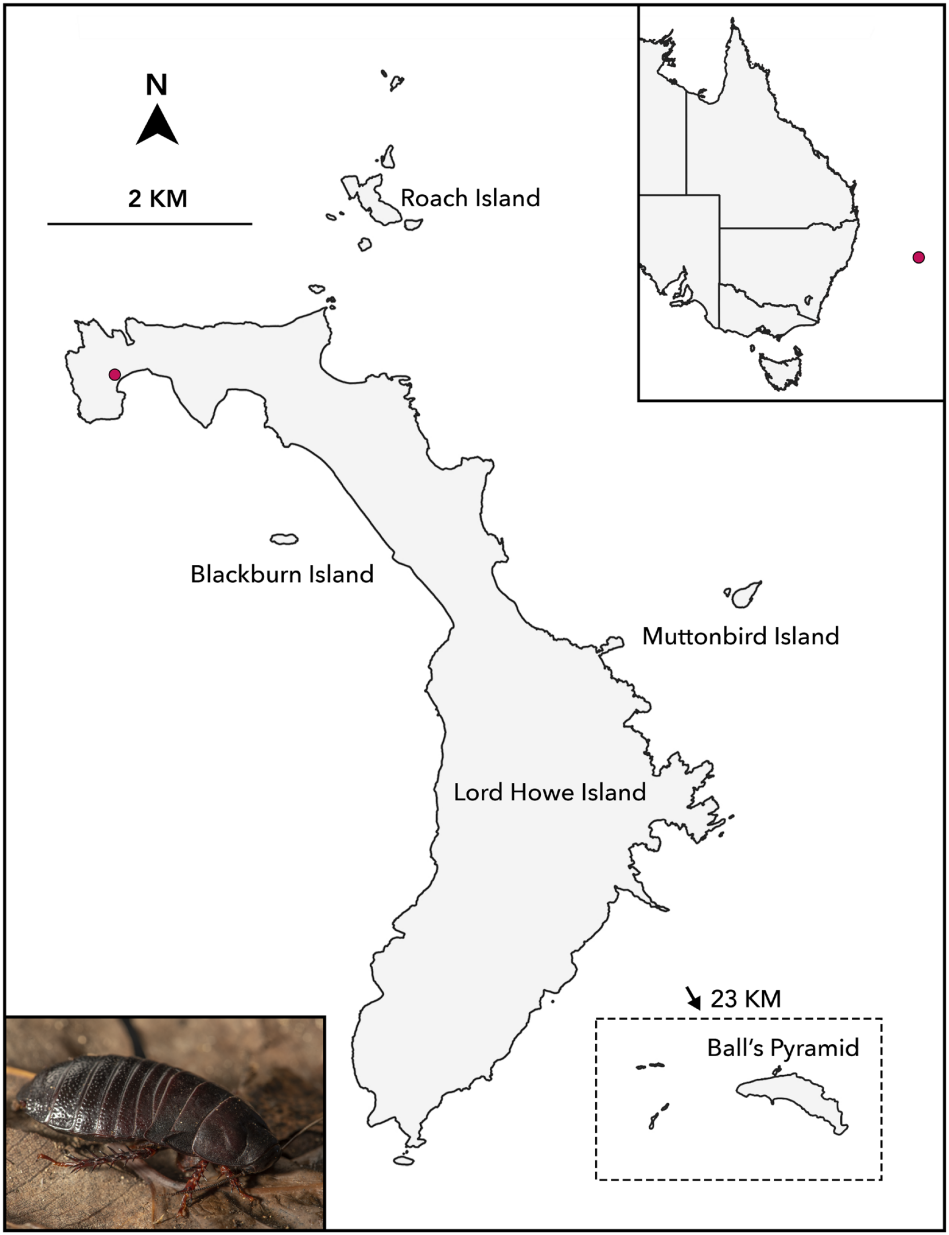
Map of the Lord Howe Island Group, showing contemporary arrangement of islands. Ball's Pyramid is the only land mass to have remained permanently isolated throughout Pleistocene sea‐level fluctuations. Marker denotes the putative relict population of *Panesthia lata* on Lord Howe Island, in North Bay. *Top right*: Position of the islands relative to eastern Australia. *Bottom left*: *P. lata* in lateral view. Photograph: J. Gilligan.

The LHIG has been subject to substantial research focusing on processes of genetic divergence in insular systems, including speciation with gene flow (e.g., Osborne et al. 2019, 2020; Papadopulos et al. 2011, 2013, 2014; Savolainen et al. 2006), cryptic population structure due to landscape heterogeneity (Major et al. 2021) and genetic diversity in small populations (Shapcott et al. 2012). Yet, to date, this work has been all but limited to LHI itself with no studies of genetic structure across > 2 islands in any species. This leaves it unknown how Pleistocene land bridges mediated migration and admixture. Almost all of the LHIG's islands have been episodically connected by Pleistocene land bridges, with only Ball's Pyramid remaining isolated over the long term (Rogers et al. 2023; Figure 1). Mikheyev et al. (2017) found minimal genetic differentiation (< 1%) between specimens of the stick insect *Dryococelus australis* from Ball's Pyramid and LHI, although no samples from other islets were available as comparisons.

In recent years, the question of archipelago‐wide genetic structure has gained relevance for conservation. The human‐mediated introductions of house mice ( *Mus musculus* ) in the 1860s and ship rats ( *Rattus rattus* ) in 1918 precipitated the extinctions of at least 20 endemic species, and declines of potentially hundreds more (DECC 2007; Hutton et al. 2007; Wilkinson and Priddel 2011). In 2019, after more than a decade of planning, both species were successfully extirpated from LHI, representing the largest rodent eradication ever undertaken on an inhabited island. While many prey populations have now begun to rebound, a large number remain substantially reduced and fragmented (N. Carlile, unpub. data). Importantly, all of the smaller islets remained completely rodent‐free throughout this period, serving as refugia for numerous birds, reptiles and invertebrates (Cassis et al. 2003; Reid et al. 2020). These now represent significant insurance populations and sources of propagules for conservation translocations (e.g., DECC 2007; Hutton et al. 2007). The management of the islets would be enhanced by an understanding of the evolutionary relationships between their communities and of their relative levels of genetic diversity.

One promising candidate for such a study is the critically endangered Lord Howe Island cockroach *Panesthia lata* (Walker 1868). A large (~40 mm), flightless insect, *P. lata* is an ecologically important decomposer, feeding on decaying plant material and burrowing into shallow topsoil (Carlile et al. 2018). The species has one of the broadest recorded distributions of any LHIG species, spanning LHI, Ball's Pyramid, Blackburn Island, and Roach Island, and potentially other unsurveyed islets. Previous phylogenetic studies have estimated that the Blackburn and Roach Island populations diverged either 2–5 Ma (Lo et al. 2016) or *ca*. 330 ka (Adams et al. 2025), potentially evidencing long‐term isolation over multiple sea level cycles. However, these studies lacked the geographic and molecular resolution to fully characterize genetic structure across the species’ range.

Following the arrival of ship rats, observations of *P. lata* declined precipitously on the main island (reviewed by Carlile et al. 2018). In 2004, the New South Wales Scientific Committee evaluated that the cockroach had likely become locally extinct on LHI (NSW Scientific Committee 2004), although no formal surveys have subsequently been undertaken to confirm the species' absence. The remaining populations on Roach and Blackburn islands appear small and spatially restricted (Carlile et al. 2018; Rose 2003), and little is known about the species' ecology on Ball's Pyramid.

In this study, we undertake the first population genetic analysis across multiple islands of the LHIG, utilising both contemporary and museum samples of *P. lata* from across the species' known range. Our aims are to: (1) further investigate whether *P. lata* has become locally extinct on LHI; (2) test whether genetic structure in *P. lata* reflects the historical connectivity of palaeo‐islands; and (3) characterise the genetic diversity and inbreeding levels of each population. In view of the low vagility and wide distribution of *P. lata* , we consider the species to be a promising model for investigating the biogeographic history of the LHIG and hope that our results lay the groundwork for the management of other organisms across the archipelago.

## 2 Methods

### 2.1 Taxon Sampling and DNA Extraction

Samples of *P. lata* were collected from throughout the species' known habitat range. In August and July 2022, we retrieved specimens from Blackburn Island (*n* = 15) and Roach Island (*n* = 5). Because only a small number of samples were found on Roach Island in 2023, we also included specimens collected from this location in December 2000 (*n* = 6) and December 2003 (*n* = 11), held in the Australian Museum, Sydney, and the private collection of Author 4, respectively. All samples were stored in 70–100% ethanol prior to DNA extraction.

Due to the inaccessibility of Ball's Pyramid, we sampled tissue from specimens collected in 1969 (*n* = 2), which represent the only known material from this population. We also sampled specimens that had been collected on LHI between 1869 and 1950 to allow us to characterise genetic diversity prior to rodent incursion (*n* = 20). These historical samples were sourced from the pinned collections of the Macleay Museum, Sydney and the Australian Museum, Sydney (Table S1).

Further, in July 2022, we opportunistically examined sites across LHI for the presence of *P. lata* , resulting in the discovery of a potential relict population in the island's North Bay (Figure 1). Based on coarse estimates of the population's density, we retrieved 10 specimens from the population, which represented considerably less than 10% of the total abundance and thereby avoided unnecessary risk to the population.

### 2.2 DNA Extraction and SNP Library Preparation

DNA was extracted from leg muscle tissue. For contemporary specimens (< 25 years old), we extracted DNA using a DNeasy Blood and Tissue Kit (Qiagen, Germany) per the manufacturer's instructions. The purity and concentration of each DNA sample were measured using a NanoDrop 2000 spectrophotometer (Thermo Fisher Scientific, USA) and a Qubit 2.0 fluorometer (Invitrogen, USA), respectively. For historical samples (> 25 years old), DNA extraction was undertaken at the Australian National Insect Collection, Canberra, utilising a proteinase K‐digestion and silica filter‐based approach that is suitable for fragmented DNA (see Jin et al. 2020; Zwick and Zwick 2023 for methods). In total, 69 individuals were sampled for SNP genotyping (Blackburn Island, *n =* 15; Roach Island, *n* = 22; North Bay, *n* = 10; historical LHI, *n* = 20; Ball's Pyramid, *n* = 2; Table S1).

SNP genotyping was performed by Diversity Arrays Technology (DArT), Canberra, using the DArTseq reduced‐representation sequencing platform (following methods in Cruz et al. 2013; Kilian et al. 2012). Briefly, genome complexity was reduced by digestion with the *PstI* and *HpalI* restriction enzymes, then sequenced on a NovaSeq 6000 S1 with 138‐bp single‐end reads. The resultant short‐read sequences were processed through the DArT analytical pipeline, which removes poor‐quality sequences, demultiplexes reads, and subsequently calls SNPs using the proprietary DArTsoft14 algorithms. SNPs were called against a reference genome of the closely related species *Panesthia cribrata* (Ewart et al. 2024). Due to DNA degradation, no historical LHI samples were successfully genotyped.

SNP data were filtered primarily using the R package *dartR* v.2.7.2 (Gruber et al. 2018) in RStudio (RStudio Team 2020), under varying criteria to suit different analyses. First, we removed SNPs that were potentially erroneous or that had high levels of missing data, based on reproducibility (< 100%), minor allele frequency (MAF < 0.03; based on 3/2 *N*) and call rate (< 0.8). Individuals with high levels of missing data (call rate < 0.1) were also removed. This initial (‘quality‐controlled’) data set comprised 10,370 SNPs from 48 individuals.

Second, the quality‐controlled SNPs were further filtered to meet the assumptions of specific analyses, which require unlinked and neutral markers. Using *dartR*, we removed individuals with a more stringent missing data threshold of 20% (i.e., call rate < 0.8), thereby excluding both samples from Ball's Pyramid and two samples from Roach Island. We then removed monomorphic loci and retained one randomly selected SNP per sequence tag to reduce linkage between markers (removed ‘secondaries’). Identification and removal of loci deviating from Hardy–Weinberg equilibrium (HWE) was performed with all samples combined, using the exact test and an alpha value of 0.01, calculated with the mid‐p method. We also checked for loci potentially under selection using BayeScan v.2.1 (Foll and Gaggiotti 2008), which detects outlier SNPs with significantly elevated pairwise *F*_ST_ values. Following the recommendations of the package, BayeScan was run using all individuals for 500,000 steps with prior odds of 100, 20 pilot runs and a 10% burn‐in. No outliers were detected at the false discovery rate threshold of *q* = 0.05. This (‘neutral’) data set comprised 8012 SNPs from 44 individuals. In addition, while filtering for conformation to HWE across all samples can be a conservative and important step to ensure adherence to statistical assumptions of analyses (e.g., Ewart et al. 2021; Gruber et al. 2018; Hendricks et al. 2018), it can also artefactually deflate inferred levels of population structure (Pearman et al. 2022). We therefore generated a third SNP panel via the same steps as the neutral data set except without HWE filtering (the ‘HWE‐unfiltered’ data set), with which we repeated certain population structure analyses. Mean coverage for the quality‐controlled, neutral and HWE‐unfiltered data sets was 11.82×, 11.85× and 11.67×, respectively (Table S2).

### 2.3 Mitogenomic Data

We previously generated partial and complete sequences of mitochondrial genomes (‘mitogenomes’) from a subset of the same specimens of *P. lata* sampled for SNP generation, encompassing all contemporary and historical populations (Adams et al. 2025; note that the Roach Island samples were all drawn from the 2000 sampling collection). These were combined with mitogenomic sequences from the closely related outgroup species *P. cribrata* and *Panesthia matthewsi*. All data were sourced from GenBank (Table S1). We excluded intergenic regions and aligned each gene individually using MUSCLE (Edgar 2004). We checked for reading frames and premature stop codons in Seqotron v.1.0.1 (Fourment and Holmes 2016), and removed ambiguously aligned regions. Our final alignment totalled 14,592 bp across 21 samples.

### 2.4 Population Structure

We employed three different methods to assess population structure based on the SNP data set. Before proceeding, we checked whether allele frequencies in Roach Island varied between time points by visualising intra‐population structure with a principal coordinates analysis (PCoA) and calculating pairwise kinship between all individuals (see below for methods). No discernible pattern was found, and all samples were retained for analysis (Figure S1).

First, to visually summarise patterns of genetic variation, we conducted a PCoA of individual allele frequencies in *dartR* using the quality‐controlled data. Before proceeding, we applied a stringent filter for call rate (> 0.95) to account for a high proportion of missing data in the Ball's Pyramid samples. Remaining analyses were performed using the neutral data set (omitting both individuals from Ball's Pyramid).

Second, we performed a Bayesian clustering analysis in STRUCTURE v.2.3.4 (Pritchard et al. 2000), automated and parallelized with STRAUTO v.1.0 (Chhatre and Emerson 2017). We modelled up to six ancestral populations (*K* = 1–6), with 10 replicates for each value of *K*, no prior population information, and assuming admixture and independent allele frequencies. Each replicate was run for 5,000,000 steps with a burn‐in of 10%. The optimal *K* value was subsequently selected as the modal value across six different metrics calculated in StructureSelector (Figure S2; Li and Liu 2018). We validated the results using a hierarchical approach, whereby the clusters identified in this step were separated, refiltered and re‐analysed independently. The results of replicate runs were summarised and visualised using CLUMPAK (Kopelman et al. 2015) implemented in StructureSelector. To ensure the results were not biased by inbreeding (Frichot et al. 2014), we also performed a population structure analysis using a sparse non‐negative matrix factorization algorithm (sNMF) in the R package LEA v.2.0 (Frichot and François 2015). We searched for the optimal number of populations between *K* = 1 and *K =* 6, with 10 replicates for each *K* value.

Third, we estimated genetic divergence between populations by calculating pairwise *F*_ST_ values (Weir and Cockerham 1984) with the R package *StAMPP* v.1.6.3 (Pembleton et al. 2013). We tested statistical significance with 95% confidence intervals constructed using 10,000 permutations.

To check whether the observed population structure was influenced by the presence of closely related individuals, we calculated pairwise individual kinship coefficients using maximum‐likelihood estimation in the R package *SNPRelate* v.1.33.0 (Zheng et al. 2012). We removed one sample from each pair with kinship ≥ 0.125 and re‐ran the PCoA, STRUCTURE, and *F*_ST_ analyses. Following the removal of relatives, the raw SNP data were filtered with criteria as described above, including a call rate filter of > 0.95 for the analysis including Ball's Pyramid samples. Further, we repeated the clustering analyses and *F*_ST_ estimation using the HWE‐unfiltered data.

### 2.5 Genetic Diversity

We investigated levels of genetic diversity within the North Bay, Blackburn Island and Roach Island populations. Because the temporally disjunct sampling of Roach Island may have led to biased estimates of inbreeding, we first ran all analyses using the complete data sets, then repeated the procedure using only the 11 Roach Island individuals collected in 2003 (representing the largest single sample). The data were re‐filtered using methods as above. In addition, to examine the effects of coverage upon diversity estimates, we ran the analyses a third time using only the loci with coverage ≥ 10×.

We quantified genetic diversity based on the neutral SNP data set by calculating observed and expected heterozygosity, as well as allelic richness, using *hierfstat* v.0.5–11 (Goudet 2005) and *PopGenReport* v.2.2.2 (Adamack and Gruber 2014), respectively. Allelic richness was measured with rarefaction to account for uneven sample sizes. To measure inbreeding, we calculated *F*_IS_ values in *hierfstat* and produced 95% confidence intervals in base R with 10,000 permutations. We also estimated kinship coefficients (Loiselle et al. 1995) for each pair of individuals in GenoDive, then averaged these values within and between populations. This method differs from that used in *SNPRelate* by including self‐kinship, which is valuable in population diversity estimates (Frankham et al. 2017).

Finally, we estimated effective population size (*N*_e_) from quality‐controlled SNPs using the linkage disequilibrium method in Ne‐Estimator v.2.1 (Do et al. 2014). Corrections were applied for sampling bias (Waples 2006) and missing data (Peel et al. 2013). We ran the analysis assuming random mating and report 95% confidence intervals constructed using the jack‐knife method, which is more appropriate for large SNP panels (Jones et al. 2016).

### 2.6 Phylogenetic Analysis

Phylogenetic analyses were undertaken using the mitogenomic data set. In line with previous work on *Panesthia* cockroaches (see Adams et al. 2025; Beasley‐Hall et al. 2021), we divided the data into six partitions: *CO1*, first, second and third codon positions of remaining protein‐coding genes, rRNAs, and tRNAs. *CO1* was assigned a separate partition (not segregated by codon position) to enable molecular clock calibration (see below), and preliminary analyses confirmed that its segregation did not influence phylogenetic inference. We estimated the maximum‐likelihood tree topology in IQTREE2 v.2.2.2 (Minh et al. 2020), using the inbuilt ModelFinder function (Kalyaanamoorthy et al. 2017) to infer the optimal substitution model for each partition based on Bayesian information criterion scores (Table S3). Node support was estimated using 10,000 ultrafast bootstrap replicates (UFBoot; Hoang et al. 2018) and the SH‐like approximate likelihood‐ratio test (SH‐aLRT).

We jointly estimated phylogenetic relationships and the evolutionary timescale using a Bayesian framework in BEAST v.1.10.4 (Suchard et al. 2018), with a separate GTR + I + G model applied to each partition, which was closest to the models estimated by ModelFinder. In the absence of any appropriate biogeographic or fossil calibrations, we opted to specify an informative prior for the substitution rate of *CO1*. We performed two separate analyses, using the highest and lowest published estimates of molecular rates in insects that diversified during or immediately after the Pleistocene epoch. To obtain a maximum age estimate, in the first analysis we specified a uniform prior of 2.35–3.15 × 10^−2^ substitutions/site/Myr on the rate, based on the dispersal of psyllid plant lice to La Palma Island following its formation *ca*. 2 Ma (Percy et al. 2004). To obtain a minimum age estimate, in the second analysis we specified a uniform prior of 1.44–1.73 × 10^−1^ substitutions/site/Myr on the rate, based on the radiation of *Neoconocephalus lyristes* crickets following the opening of the St. Lawrence Seaway *ca*. 11 ka (Ney et al. 2018). In both analyses, we maximised the models' adherence to the rate calibrations by implementing a strict molecular clock, and we compared coalescent (constant size) and birth‐death tree priors. While the insects used for the calibrations represent the most closely related organisms with molecular rate estimates in the same approximate timescale of diversification (Ney et al. 2018), they are nonetheless phylogenetically distant from *Panesthia lata*, which introduces uncertainty into our date estimates. However, the choice of a more phylogenetically proximal, but temporally distant, calibration would have been likely to more substantially confound date estimates, potentially over‐estimating node ages by orders of magnitude (Ho et al. 2011). Two replicate runs of 2 × 10^7^ Markov chain Monte Carlo steps were performed for each analysis, drawing samples every 10^4^ steps. Sufficient sampling after convergence was checked using TRACER v.1.7.2 (Rambaut et al. 2018), and maximum‐clade‐credibility trees were generated in TreeAnnotator with a 10% burn‐in.

In supplement to the phylogenetic analyses, we produced a 4‐gene alignment comprising partial sequences of *CO1*, *CO3*, *NAD1* and *NAD5* with no missing nucleotides (2305 bp, *n* = 16; two samples from historical LHI and the single sample from Ball's Pyramid were excluded due to high levels of missing data). This was used to estimate a haplotype network using the statistical parsimony TCS method (Clement et al. 2002) in PopART (Leigh and Bryant 2015).

To check for congruence in the phylogenetic signals between the mitogenomes and nuclear data, we also undertook a phylogenetic analysis of the SNP data set. We filtered the quality‐controlled data set for call rate (> 0.95) and secondaries in *dartR*, then concatenated the SNPs to produce an alignment of 1315 sites. Heterozygous loci were assigned standard ambiguity codes. Following recommendations of the package, we inferred the phylogeny using maximum likelihood in IQTREE2 with a GTR + I + G substitution model. Based on results from PCoA analyses, the root was placed between the samples from Ball's Pyramid and all other taxa. For robustness, we repeated the analysis without the Ball's Pyramid samples, as these were highly divergent from the other populations (re‐filtering the data as above to produce an alignment of 3754 bp).

## 3 Results

### 3.1 Genetic Structure

**FIGURE 2:**
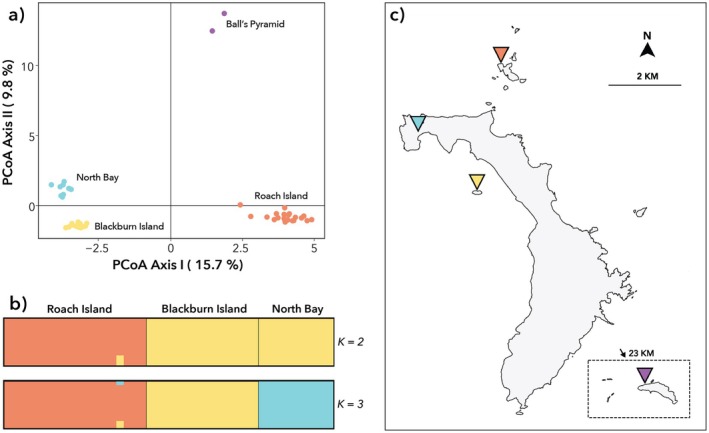
(a) Principal coordinates analysis (PCoA) plot of 48 samples of *Panesthia lata*, using 1366 single‐nucleotide polymorphisms (SNPs). (b) STRUCTURE plots for 44 samples based on 8012 SNPs, when *K* = 2 and *K* = 3 (samples from Ball's Pyramid were excluded from this analysis). (c) Map of the Lord Howe Island Group, with labels coloured accordingly to PCoA clusters.

PCoA partitioned genetic variation into four discrete clusters, corresponding to the four contemporary island populations (Figure 2a). In the output plot, cockroaches from Ball's Pyramid and Roach Island were distant from those from Blackburn Island and North Bay, with Ball's Pyramid in turn somewhat distant from Roach Island.

Relationships between the LHI palaeo‐island populations (i.e., excluding samples from Ball's Pyramid) were investigated using sequential STRUCTURE analysis. This suggested that genetic variability was optimally explained by *K* = 2–3 clusters, depending on the estimator, with most supporting *K* = 3 (Figure S2). For all values of *K* ≥ 3, there was clear genetic differentiation between the samples from each of the three islands; however when *K* = 2, the samples from North Bay and Blackburn Island collapsed into a single cluster (Figure 2b). Admixture between the populations was negligible irrespective of the *K* value. When samples from each island were analysed separately, no additional population structure was evident. sNMF analysis also supported *K* = 3, with slightly higher proportions of admixture (Figure S3). In concordance, pairwise *F*_ST_ values evidenced high, and significant, genetic differentiation between the three populations, with the greatest distance between Roach Island and North Bay (Table 1).

**TABLE 1:** Genetic differentiation between and within populations of *Panesthia lata* based on 8012 SNPs.

	Roach Island	Blackburn Island	North Bay
Roach Island	**0.199**	−0.105	−0.116
Blackburn Island	0.239* (0.229–0.250)	**0.202**	−0.011
North Bay	0.279* (0.270–0.289)	0.225* (0.216–0.234)	**0.328**

*Note:* Below diagonal: Pairwise *F*_ST_ values (95% confidence interval) (* denotes values significantly different from 0). On diagonal: Average pairwise kinship within populations. Above diagonal: Average pairwise kinship between populations.

Following the removal of closely related individuals, the data set comprised 12 samples from Blackburn Island, 19 samples from Roach Island, and 2 samples from North Bay. PCoA clusters, STRUCTURE clusters, and pairwise *F*_ST_ values estimated from this data set were similar to those found using all samples (Figure S4; Table S4). *F*_ST_ was upwardly biased by the low genetic diversity among the retained individuals (see Meirmans and Hedrick 2010). When analyses were re‐run using the HWE‐unfiltered data set, STRUCTURE and sNMF analyses both optimised at *K* = 3, with similar levels of admixture as inferred from the neutral data set (Figure S5). *F*_ST_ values estimated from this data set were similar to or slightly higher than those estimated from the neutral SNPs (Table S5).

### 3.2 Genetic Diversity

Average pairwise kinship was high within all populations, with the highest value seen in North Bay and the lowest in Roach Island (Table 1; note that a kinship of *k* = 0.25 is the average for siblings). Average pairwise kinship between all populations was negative, indicating an absence of recent gene flow; however, average kinship was lowest between the populations from Roach Island and North Bay and highest between those from Blackburn Island and North Bay.

Heterozygosity and allelic richness were highest in the cockroaches from Roach Island and lowest in North Bay, although the differences were relatively modest (Table 2). Further, North Bay was estimated to have a substantially smaller effective population size than Roach Island (Table 2), albeit with broad confidence intervals. The effective population size of Blackburn Island could not be estimated, likely due to insufficient sample size (Do et al. 2014). Estimates of *F*_IS_ were significantly above 0 in all populations, indicating significant deviation from panmictic mating (Table 2). In contrast to other metrics of diversity, the estimated *F*_IS_ was highest in Roach Island and lowest in Blackburn Island. Results were similar for all metrics when analyses were repeated using only the 11 Roach Island specimens collected in 2003 (Table S5). Estimates of genetic diversity were slightly higher, and estimates of *F*_IS_ slightly lower, after filtering for high‐coverage loci, although patterns of relative diversity remained constant (Table S6).

**TABLE 2:** Summary of genetic diversity statistics across three populations of *Panesthia lata*.

Population	*N*	*A*_R_	*H*_O_ (SE)	*H*_E_ (SE)	*F*_IS_ (95% CI)	*N*_e_ (95% CI)
Roach Island	19	1.480	0.129 (0.032)	0.180 (0.038)	0.291* (0.282–0.300)	182.6 (66.1–Inf.)
Blackburn Island	15	1.395	0.120 (0.040)	0.149 (0.043)	0.201* (0.191–0.211)	Inf.
North Bay	10	1.362	0.116 (0.054)	0.145 (0.059)	0.214* (0.202–0.226)	48.9 (31.2–103.5)

*Note:* All values except *N*_e_ based on 8012 SNPs, *N*_e_ values based on 10,370 SNPs.

Abbreviations: *N*, sample size; *A*_R_, mean rarefied allelic richness; *H*_O_, mean observed heterozygosity; *H*_E_, mean expected heterozygosity; SE, standard error; *F*_IS_, inbreeding coefficient (* denotes value significantly different to 0); *N*_e_, effective population size; 95% CI, 95% lower and upper confidence interval.

### 3.3 Phylogenetic Analyses and Evolutionary Timescale

**FIGURE 3:**
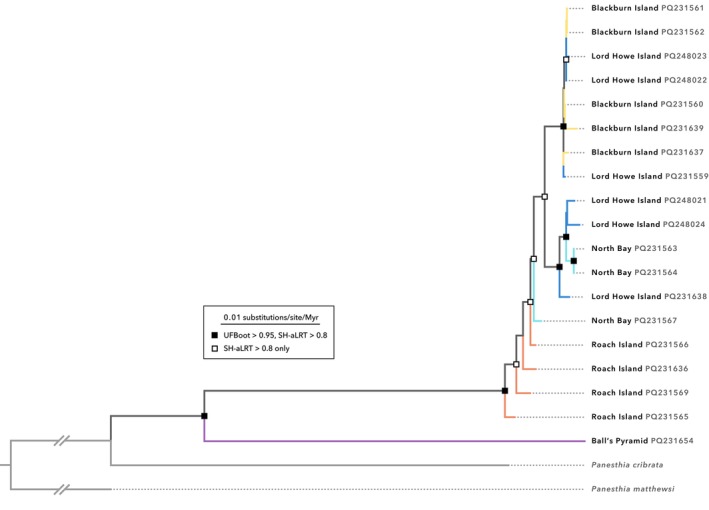
Maximum‐likelihood phylogeny of *Panesthia lata* inferred from complete mitochondrial genomes in IQTREE2. UFBoot: Ultrafast bootstrap; SH‐aLRT: SH‐like approximate likelihood‐ratio test. Numbers next to tip labels indicate GenBank accession numbers. Specimens labelled with ‘Lord Howe Island’ are historical samples collected prior to near‐extirpation by rodents.

**FIGURE 4:**
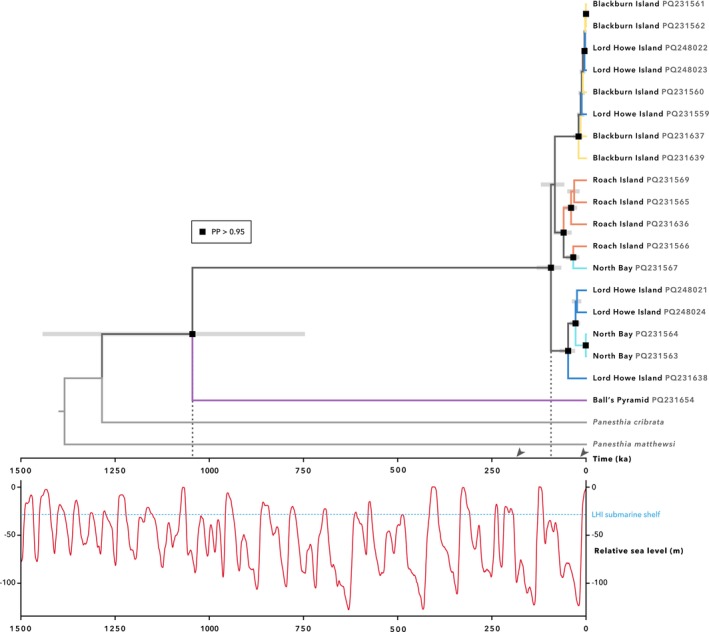
Dated phylogeny of *Panesthia lata* inferred from complete mitochondrial genomes in BEAST. The figure depicts our maximum age estimate, which was obtained by specifying an informative prior of 2.35–3.15 × 10^−2^ substitutions/site/Myr on the substitution rate of *CO1* (Percy et al. 2004). Dashed lines denote the age of the divergence of the Ball's Pyramid lineage and the crown age of the Lord Howe palaeo‐island clade, respectively. Arrows denote these ages in our minimum estimate, which was inferred using a prior of 1.44–1.73 × 10^−1^ substitutions/site/Myr (Ney et al. 2018). PP: Posterior probability. Numbers on labels indicate GenBank accession numbers. *Inset*: Relative sea level over the last 1.5 Myr, adapted from Bintanja and van de Wal (2008). Dashed line indicates the median contemporary depth of the Lord Howe palaeo‐island submarine shelf (Kennedy et al. 2002). Under both estimates, the divergence of the Lord Howe, Blackburn and Roach Island samples initiated during the most recent glacial period.

Maximum‐likelihood and Bayesian analyses of complete mitogenomes both placed the Ball's Pyramid population as the sister group to the remaining lineages with high support (UFBoot, SH‐aLRT, PP = 1; corrected p‐distance = 3.51%–4.50%; Figures 3 and 4). However, relationships between and within the other populations were inconsistent. In the maximum‐likelihood phylogeny, samples from Roach Island formed a paraphyletic grade with respect to those from Blackburn Island, North Bay and historical LHI, albeit with equivocal node support (Figure 3). Individuals from Blackburn Island and North Bay formed two reciprocally monophyletic clades, although both were nested among historical LHI samples, once more with relatively poor node support.

Bayesian analysis yielded a contrasting topology, inferring a closer relationship between cockroaches from Blackburn Island and Roach Island (Figure 4). With the exception of the individuals from Ball's Pyramid, none of the populations were recovered as monophyletic. Individuals from North Bay and Blackburn Island once more grouped with historical LHI specimens, with variable node support. Curiously, the Roach Island samples formed a well‐supported clade that was paraphyletic with respect to a single individual from North Bay. Using a low and high estimate of the substitution rate for CO1, we obtained estimates of divergence times that we consider to be maximum and minimum values, respectively. Using the lower estimate of the substitution rate, the cockroaches from Ball's Pyramid were inferred to have diverged from the remaining populations *ca*. 1.04 Ma (95% highest posterior density interval [HPD] 0.784–1.44 Ma), while the crown age of the LHI palaeo‐island clade was found to be *ca*. 93.1 ka (95% HPD 67.4–129 ka; Figure 4). Using the higher estimate for the substitution rate yielded shallower and narrower date ranges (Figure S6). The divergence of Ball's Pyramid samples was inferred to have occurred *ca*. 185 ka (95% HPD 133–242 ka), and the crown age of the LHI palaeo‐island was estimated at *ca*. 16.5 ka (95% HPD 12.0–21.8 ka). These estimates were derived using a coalescent tree prior, and values were nearly identical under a birth‐death tree prior.

Eleven haplotypes were observed in the 4‐gene mitochondrial dataset. Haplotype network analysis of this dataset separated cockroaches from Roach Island from the samples from Blackburn Island, North Bay, and historical LHI, which did not form discrete clusters (Figure S7).

**FIGURE 5:**
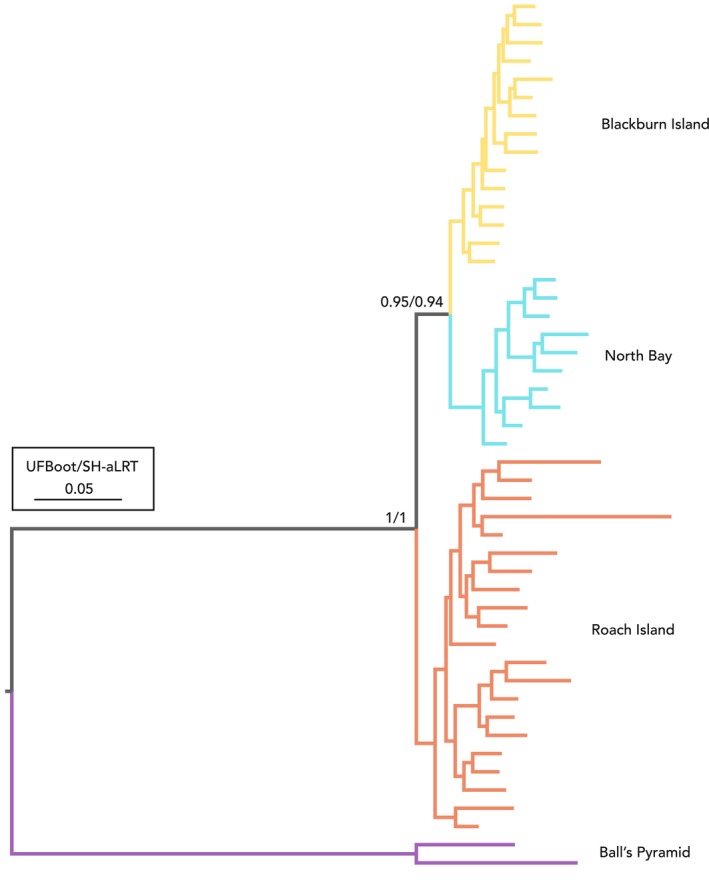
Maximum‐likelihood phylogeny of *Panesthia lata* inferred from 1315 nuclear SNPs in IQTREE. UFBoot: Ultrafast bootstrap, SH‐aLRT: SH‐like approximate likelihood‐ratio test. Scale bar represents substitutions/site/Myr. The root was placed between samples from Ball's Pyramid and remaining populations. Node support within populations is not shown.

Finally, maximum‐likelihood analysis of nuclear SNPs resolved all populations as reciprocally monophyletic, with or without the inclusion of the Ball's Pyramid samples (Figure 5; Figure S8). When rooted with Ball's Pyramid samples, the samples from Roach Island formed a well‐supported sister group to those from Blackburn Island and North Bay (UFBoot, SH‐aLRT = 1).

## 4 Discussion

### 4.1 Phylogeography and Sea Level

The pattern of genetic structure in *P. lata* is consistent with the geographic arrangement of Pleistocene palaeo‐islands. Across all analyses of mitochondrial and nuclear data, the population on Ball's Pyramid was especially divergent from the remaining samples, reflecting its permanent geographic isolation from the LHI palaeo‐island (Gilmore et al. 2020; McDougall et al. 1981). While we acknowledge a higher proportion of missing data for the Ball's Pyramid specimens, our results were robust to data filtering, indicating that the population's distinctiveness was not artefactual. In concordance, our divergence date estimates range from 185 ka to 1.04 Ma, suggesting that the population has been isolated over multiple sea‐level cycles. We therefore provide to our knowledge the first genetic evidence of the evolutionary distinctiveness of the Ball's Pyramid fauna (Flemons et al. 2018; Lillemets and Wilson 2002). This result contrasts with the low sequence divergence between LHI and Ball's Pyramid samples of *D. australis* (Mikheyev et al. 2017), although it is reasonable for the timing of island colonisation to differ across resident species.

The weaker differentiation among the remaining populations points to a more recent divergence, presumably related to land‐bridge formation during sea‐level lowstands. We cannot completely exclude the possibility of human‐mediated transport; however, phenotypic differences between the cockroaches from each population suggest that isolation has exceeded the period of human settlement (*ca*. 200 years). Moreover, our time‐calibrated phylogenies indicate that the LHI palaeo‐island populations began to diverge between 16.6 and 93.1 ka. Due to the non‐monophyly of mitochondrial lineages and the recency of isolation, it is challenging to achieve precise molecular date estimates, and such molecular ages are likely to be older than the age of geographic separation (Edwards and Beerli 2000; Giarla and Esselstyn 2015). However, this approximate timescale places the genetic coalescence during the previous glacial period (100–10 ka; Spratt and Lisiecki 2016), suggesting that these contemporary lineages were connected by migration into one larger population across the LHI paleo‐island before this point.

The precise mechanisms by which this larger, panmictic population began to differentiate are difficult to untangle. A parsimonious explanation, consistent with our minimum age estimate, is that the contemporary populations were isolated by sea level rise *ca*. 10 ka (e.g., Chen et al. 2012; Miranda et al. 2017; Naydenov et al. 2014). Roach Island individuals were relatively distant from North Bay and Blackburn Island in nuclear SNP analyses, as well as in the maximum‐likelihood mitochondrial phylogeny. The channel isolating Roach Island is deeper and broader than that between LHI and Blackburn Island, which further implicates sea‐level inundation as a key driver of genetic structure in *P. lata* . However, our maximum molecular date estimate supports an alternative scenario whereby divergence initiated prior to end‐Pleistocene sea‐level rise. In which case, genetic structure could have been shaped by other barriers to gene flow, such as the heterogeneous topography and/or biotic landscape of LHI (Hyman et al. 2023; Major et al. 2021). In either case, our results contrast with suggestions of sustained isolation and in situ speciation on the islets (Cassis et al. 2003; Lillemets and Wilson 2002). Instead, they indicate that Pleistocene land bridges either eroded previous genetic differentiation in *P. lata* (if isolated populations were present on multiple islets in the previous glacial period), or alternatively permitted colonisation of multiple islets (e.g., Barker et al. 2012; Cibois et al. 2010; Fattorini 2010; Fernández‐Palacios et al. 2015; Heaney et al. 2005; Mairal et al. 2015; Papadopoulou and Knowles 2017).

Our estimated evolutionary timescale stands in sharp contrast to previous estimates of the age of the Blackburn and Roach Island populations (*ca*. 3 Ma in Lo et al. 2016; *ca*. 330 ka in Adams et al. 2025). Several additional lines of evidence support the shallow timescale inferred presently. First, the dates inferred by Lo et al. (2016) and Adams et al. (2025) were based on deep fossil calibrations and a substitution rate calibrated on a more ancient timescale, respectively (and with only two samples of *P. lata* included in the phylogeny of Lo et al. 2016). It is now well established that temporally distant calibrations can lead to substantial overestimation of population‐level coalescence times (Grant 2015; Ho et al. 2011). Second, the non‐monophyly in our mitogenome analyses, even when discounting the historical LHI specimens, is more likely to reflect incomplete lineage sorting following recent rather than ancient isolation (e.g., Brüniche‐Olsen et al. 2021; Li et al. 2020). Third, while genetic studies across the LHIG are scarce, a previous phylogenetic analysis found minimal differentiation between individuals of the skink *Oligosoma lichenigerum* from Blackburn Island and LHI based on several nuclear and mitochondrial markers (Chapple et al. 2009). Taken together, these and our results support the hypothesis that recent glacial land bridges have allowed for overland migration across the LHI paleo‐island in the last 100 kyr. However, due to the wide interval between our maximum and minimum age estimates, there remains considerable uncertainty around the precise mechanisms of dispersal and isolation in *P. lata* . In future, molecular dating could be refined by evidence such as a species‐specific estimate of mutation rate.

### 4.2 Genetic Structure and Relict Populations

Our nuclear SNP analyses reveal four contemporary genetic clusters within *P. lata* , corresponding to the four islands that compose its archipelagic range. Most significantly, the genetic distinctiveness of the North Bay population confirms its status as a relic of the former diversity on LHI, as opposed to a recent transplant from another islet. By accounting for closely related individuals in PCoA and STRUCTURE analysis, we ensured that this result was not an artifact of high kinship (e.g., Ewart et al. 2021). That the population survived more than a century of rodent activity is remarkable, even if its size and spatial extent are highly limited. One possible explanation is that predation was limited by rodent control in an adjacent *Howea* palm plantation, where baiting and trapping have been underway since the 1940s (Harper et al. 2020; D. Hiscox, pers. comm.). Moreover, since our surveys were non‐exhaustive, the persistence of *P. lata* at this site raises the possibility of further relict populations being discovered in future, as the species recovers following predator release.

Despite their clear partitioning based on nuclear SNPs, the cockroaches from Roach Island, Blackburn Island and North Bay all emerged as non‐monophyletic in mitogenomic analyses. Considering the recency of divergence, this discordance could be attributable to introgression, selection upon mitochondrial variants or incomplete lineage sorting. It is not unusual for genomic SNP panels to detect fine‐scale population structure not yet manifest in the genealogies of individual loci (e.g., McCartney‐Melstad et al. 2018; Sturm et al. 2020; Younger et al. 2017). However, a significant factor in the mitochondrial non‐monophyly was the inclusion of historical LHI samples, which were not genotyped for nuclear SNPs. One explanation is that the pre‐rodent population on LHI comprised a wider diversity of haplotypes, some with close affinity to the other islets, which were subsequently lost as the population declined. Alternatively, while the historical specimens were all labelled as originating from ‘Lord Howe Island’, some may have been collected from Blackburn Island, which is near to the human settlement. Individuals from the two populations cannot be reliably separated by morphology, thus further sampling of nuclear markers will be needed to confirm the provenance of the ancient specimens.

### 4.3 Genetic Diversity and Conservation Implications

The discovery that *P. lata* has survived on LHI is a significant advancement in the species' conservation, yet there may still be need for future management. Genetic diversity and effective population size were low in all three sampled populations, consistent with their small geographic extent and insularity. We acknowledge that the small sample sizes and broad confidence intervals place substantial limits on interpretation. However, the combination of high pairwise kinship, significant deviations from panmixia (based on *F*_IS_) and low *N*_e_ suggest that all three sampled populations may be experiencing some degree of inbreeding. With further study, it will be possible to determine the presence and/or extent of inbreeding depression in *P. lata* , and whether active management will be necessary. Even modest increases in inbreeding have been found to substantially impact the long‐term viability of wild populations (Frankham et al. 2017; Ralls et al. 2018).

Our study has also provided the first comparison of genetic diversity across several islands of the LHIG. Across most metrics, diversity was highest among the cockroaches in Roach Island and lowest in North Bay. This pattern may reflect the extent and quality of habitat, whereby Roach Island (the largest islet after Ball's Pyramid) has been mostly undisturbed by human activity, while large sections of Blackburn Island have been deforested and LHI has been severely impacted by invasive rodents (DECC 2007; Priddel and Wheeler 2014). It is therefore curious that *F*_IS_ was highest in Roach Island, which could indicate a recent uptick in inbreeding or represent an artefact of small sample size. It is also possible that cryptic subdivision in the population inflated the estimate, although no subdivision was observed in STRUCTURE or PCoA analyses. While the genetic health of the Roach Island population warrants further study, the high kinship and excess of close relatives within the North Bay population are of particular concern and evidence a substantial population bottleneck due to rodent activity.

Prior to the discovery of the relict population, several authors had proposed a reintroduction of *P. lata* to LHI (Carlile et al. 2018; Hutton et al. 2007). Although reintroduction is now unnecessary, the vulnerability of the population raises the possibility of genetic rescue: the transplanting of propagules from elsewhere to counteract genetic erosion (Tallmon et al. 2004; Whiteley et al. 2015). Typically, due to the risk of outbreeding depression, genetic rescues are undertaken between populations that have been isolated for under 500 years (Frankham et al. 2011). However, this threshold is likely to be conservative (Ralls et al. 2018), and studies evidence long‐term fitness increases even when populations diverged at a comparable or earlier age than *P. lata* (Aitken and Whitlock 2013; Harrisson et al. 2016; Kronenberger et al. 2017; Pekkala et al. 2012; Weeks et al. 2017). Nonetheless, further data will be needed to assess the risk of outbreeding depression before any action is taken, including captive‐crossing or comparative genomic studies.

Finally, we confirm the presence of *P. lata* on Ball's Pyramid, which is not currently recognised in the species’ management plan due to the islet's inaccessibility for survey. Based on its significant divergence in nuclear allele frequencies and evolutionary distinctiveness (inferred from phylogenetic analysis), the population could qualify as an evolutionarily significant unit (ESU; an independently evolving, divergent unit of genetic variance; Moritz 1994; Zink 2004). ESU status could grant legal protection and encourage focused management of the unique habitat (reviewed by Funk et al. 2012). The NSW Threatened Species Scientific Committee, which has managed the conservation status of *P. lata* to date, has previously recognised ESUs based on genetic divergence (e.g., NSWTSSC 2003). If more urgent action were required, recognition under the IUCN Red List could also offer a prompter, ‘interim’ solution. While we were unable to measure genetic diversity on Ball's Pyramid, ecological observations suggest that *P. lata* has a limited presence on the island and may therefore be vulnerable to local extinction (N. Carlile, pers. obs.). Overall, our findings reveal that the natural and anthropogenic fragmentation of the LHIG has significantly impacted genetic diversity in *P. lata* , and have offered avenues for future management.

## 5 Conclusions

This study represents the first detailed, species‐wide genetic analysis across > 2 islands of the LHIG. Our results demonstrate that the LHIG is another archipelago that conforms to the traditional model of palaeo‐island biogeography, whereby genetic structure within *P. lata* reflects the proximity and historic fusion of some islets into a larger landmass, allowing for dispersal between now‐isolated land masses during the last interglacial period. From a conservation standpoint, our discovery of a highly evolutionary distinct lineage on Ball's Pyramid reiterates the need to conserve the islet's unique habitat and fauna, which appear to have been long isolated from LHI. Moreover, the relatively shallow divergence of the Lord Howe, Blackburn and Roach Island populations, alongside the potential inbreeding in these populations, provides evidence that genetic rescue between these islands could be an effective conservation strategy for *P. lata* . Though further work will be needed to confirm whether these findings can be generalised, we suggest that these results could also inform the management of other species with similar distributions across the LHIG.

## Author Contributions

**Maxim W. D. Adams:** conceptualization (equal), data curation (equal), formal analysis (equal), investigation (equal), methodology (equal), visualization (lead), writing – original draft (lead), writing – review and editing (lead). **Kyle M. Ewart:** data curation (equal), methodology (equal), writing – review and editing (equal). **Nicholas Carlile:** investigation (equal), methodology (equal), supervision (equal), writing – review and editing (equal). **Harley A. Rose:** investigation (equal), supervision (equal), writing – review and editing (equal). **James A. Walker:** investigation (equal), methodology (equal), writing – review and editing (equal). **Ian Hutton:** investigation (equal), writing – review and editing (equal). **Simon Y. W. Ho:** data curation (equal), software (equal), supervision (equal), writing – review and editing (equal). **Nathan Lo:** conceptualization (equal), investigation (equal), methodology (equal), supervision (equal), writing – review and editing (equal).

## Funding

This work was supported by the Australia and Pacific Science Foundation (grant APSF22029).

## Conflicts of Interest

The authors declare no conflicts of interest.

## Supporting information


**Appendix S1:** Supporting Information.

## Data Availability

Raw single‐nucleotide polymorphism data, phylogenetic trees and tables have been deposited on Dryad, DOI: 10.5061/dryad.c2fqz61m0.
